# Correction: Behavior-change interventions to improve antimicrobial stewardship in human health, animal health, and livestock agriculture: A systematic review

**DOI:** 10.1371/journal.pgph.0002930

**Published:** 2024-02-05

**Authors:** Jessica Craig, Aditi Sriram, Rachel Sadoff, Sarah Bennett, Felix Bahati, Wendy Beauvais

## Notice of republication

This article was republished on January 29^th^, 2024, to correct the author list and add Aditi Sriram as the second author. Please download this article again to view the correct version. The originally published, uncorrected article and the republished, corrected article are provided here for reference.

## Supporting information

S1 File Originally published, uncorrected article. (PDF)

S2 File Republished, corrected article. (PDF)

## References

[1] Craig J, Sriram A, Sadoff R, Bennett S, Bahati F, Beauvais W (2023) Behavior-change interventions to improve antimicrobial stewardship in human health, animal health, and livestock agriculture: A systematic review. PLOS Glob Public Health 3(5): e0001526. doi: 10.1371/journal.pgph.0001526 37155592 PMC10166487

