# Correction: Behavior-change interventions to improve antimicrobial stewardship in human health, animal health, and livestock agriculture: A systematic review

**DOI:** 10.1371/journal.pgph.0004856

**Published:** 2025-06-24

**Authors:** Jessica Craig, Aditi Sriram, Rachel Sadoff, Sarah Bennett, Felix Bahati, Wendy Beauvais

An additional affiliation is missing for the first, third to sixth authors. Jessica Craig, Rachel Sadoff, Sarah Bennett, and Felix Bahati are also affiliated with One Health Trust, Washington, DC, United States of America.
