# Supplementary material for: Behavior-change interventions to improve antimicrobial stewardship in human health, animal health, and livestock agriculture: A systematic review
Source: PLOS Glob Public Health. 2023 May 8;3(5):e0001526. doi: 10.1371/journal.pgph.0001526 (PMC10166487; doi:10.1371/journal.pgph.0001526)
Supplement: Attachment — Submitted filename: Response to reviewers.docx [file pgph.0001526.s006.docx]

Response to reviewers

We thank the editorial board and the reviewers for their thoughtful review and feedback on our manuscript titled, “Behavior-change interventions to improve antimicrobial stewardship in human health, animal health, and livestock agriculture: A systematic review.”

Please find below a summary table that lists each question or comment raised by the reviewer, our response to the question or comment, and the location of the changes made to address it. In addition to the changes noted below, we added an additional reference (#27) on page 7 which resulted in a shifting in all subsequent reference numbers.

| Reviewer Num. | Reviewer Comment/Question | Author Response | Location of Changes to Address Reviewer Comment/Question |
| --- | --- | --- | --- |
| Reviewer 1 | L127: What is 'risk ratio'? What is it? It is only "Odd ratio' or relative risk'. Delete it. | Per the US CDC, the definition of a risk ratio is as follows: “A risk ratio (RR), also called relative risk, compares the risk of a health event (disease, injury, risk factor, or death) among one group with the risk among another group. It does so by dividing the risk (incidence proportion, attack rate) in group 1 by the risk (incidence proportion, attack rate) in group 2. The two groups are typically differentiated by such demographic factors as sex (e.g., males versus females) or by exposure to a suspected risk factor (e.g., did or did not eat potato salad). Often, the group of primary interest is labeled the exposed group, and the comparison group is labeled the unexposed group.”^1^ For papers included in our study that reported the risk of a specific event in an intervention or experimental group compared to the risk of the same event in a control or another intervention group, it was necessary to compute the risk ratio. Therefore, we will not delete this from our paper. | We clarify why such measures of association were used on page 7 and add a reference that further describes the epidemiological methodologies. |
|  | L148: Delete 'rate ratio'.  Also, check and correct similar wrongly used statistical phrases. | Per the US CDC, the definition of a rate ratio is as follows: “A rate ratio compares the incidence rates, person-time rates, or mortality rates of two groups. As with the risk ratio, the two groups are typically differentiated by demographic factors or by exposure to a suspected causative agent. The rate for the group of primary interest is divided by the rate for the comparison group.”^1^ For papers included in our study that reported data as a rate (for instance antimicrobial use as daily defined doses per 1,000 patient-days), it was necessary to compute and report the rate ratio as opposed to an Odds ratio. Therefore, we retain the rate ratios in our paper. Upon further review, we do not find any other “wrongly used statistical phrases.” | We clarify why such measures of association were used on page 7 and add a reference that further describes the epidemiological methodologies. |
| Reviewer 2 | Background: This section can be shortened. It is non-controversial at this point that non-pharmaceutical/behavioral interventions to reduce antibiotic use are increasingly necessary. | Given the global scope and intended global and multi-sectoral audience of this paper, we would like to retain this information to emphasize the importance of non-pharmaceutical/behavioral interventions. While we agree that it is non-controversial at this point, the importance of non-pharmaceutical/ behavioral interventions are often overlooked and, at this point, are not consistently addressed by facility- or national-level research, policy, and interventions efforts. | No changes made. |
|  | In line 67, the authors write that in the past 50 years no new antimicrobial agents active against Gram-negatives have been introduced in the last 50 years. This is simply not true – looking back at the document they cite, it seems that they meant to say that no new _classes_ of antibacterial active against Gram-negatives have been developed, which is true. | We agree. | We have corrected this statement on page 4 to read, “…no new classes of antimicrobial agents…” |
|  | The word “Gram” should be capitalized. | We agree. | We have capitalized this letter on page 4. |
|  | “gentamicin” is spelled incorrectly throughout. | We agree. Thank you for spotting this. | The spelling has been corrected throughout. |
|  | I think that another conclusion that could be drawn from this review is that given the sheer diversity of contexts in which antibiotics are used, the diversity of interventions, and the heterogeneity of implementations of the same intervention, that perhaps this is an impossible aspiration. And if that’s the case, the question becomes what would be an alternative way to approach the development and reporting of stewardship interventions. If the authors trim some of their introduction, it might be worthwhile to expand on that idea in the discussion, but this is optional. | This is a great suggestion and we incorporate this into our revisions. | We have added this to our discussion on pages 19 and 20. |
| ^1^U.S. CDC. (2012). Section 5: Measures of Association. Accessed 4/12/2023. Available from: <https://www.cdc.gov/csels/dsepd/ss1978/lesson3/section5.html> | | | |
